# Milk Consumption Patterns and Gut Microbiota Composition in Healthy Adults: Insights from a Propensity Score-Matched Cross-Sectional Study

**DOI:** 10.3390/nu18193272

**Published:** 2026-10-05

**Authors:** Giada La Placa, Paola Gualtieri, Daniele Peluso, Daniele Marcoccia, Giuseppe Merra, Antonino De Lorenzo, Antonella Smeriglio, Domenico Trombetta, Laura Di Renzo

**Affiliations:** 1 PhD School of Applied Medical-Surgical Sciences, Tor Vergata University of Rome, 00133 Rome, Italy; laplacagiada@gmail.com (G.L.P.); daniele.peluso@uniroma2.it (D.P.); 2 School of Specialization in Food Science, Tor Vergata University of Rome, 00133 Rome, Italy; 3 Section of Food Science, Clinical Nutrition and Pharmaceutical Science, Department of Biomedicine and Prevention, Tor Vergata University of Rome, Montpellier 1, 00133 Rome, Italy; delorenzo@uniroma2.it (A.D.L.); laura.di.renzo@uniroma2.it (L.D.R.); 4 Department of Biology, Centro di Bioinformatica Molecolare, University of Rome “Tor Vergata”, 00133 Rome, Italy; 5 Istituto Zooprofilattico Sperimentale del Lazio e della Toscana “M. Aleandri”, Via Appia Nuova 1411, 00178 Rome, Italy; daniele.marcoccia@izslt.it; 6 Department of Chemical, Biological, Pharmaceutical and Environmental Sciences, University of Messina, Viale Ferdinando Stagno d’Alcontres 31, 98166 Messina, Italy; antonella.smeriglio@unime.it (A.S.); domenico.trombetta@unime.it (D.T.)

**Keywords:** gut microbiota, milk intake, dietary patterns, environmental contaminants, dairy consumption

## Abstract

**Aim:** Milk consumption patterns may be associated with gut microbiota composition, but observational findings can be strongly influenced by demographic and body composition differences among consumers. This observational cross-sectional study evaluated whether questionnaire-derived habitual milk consumption patterns were associated with gut microbiota composition in healthy adults by comparing non-milk consumers, bovine milk consumers, and plant-based beverage consumers. **Methods**: Baseline cross-sectional data from 265 participants were analyzed: 99 non-milk consumers, 139 bovine milk consumers, and 27 plant-based beverage consumers. Clinical, anthropometric, body composition, and microbiota data were integrated. Alpha diversity was assessed using standard diversity indices, whereas beta diversity was evaluated using Bray–Curtis distances and permutational multivariate analysis of variance. Taxon-level analyses were corrected using the false discovery rate, and pairwise propensity score matching was applied as a sensitivity analysis to evaluate the influence of measured demographic, anthropometric, and body composition imbalance. **Results**: Crude analyses showed a small beta-diversity difference between bovine milk and plant-based beverage consumers (R^2^ = 0.0119, *p* = 0.014), alongside marked differences in age, body mass index, fat mass, and impedance-derived parameters. In matched sensitivity analyses, the crude beta-diversity difference between bovine milk consumers and plant-based beverage consumers was not reproduced in a smaller matched subset (pair-stratified PERMANOVA R^2^ = 0.0214, *p* = 0.506). No matched comparison showed significant alpha-diversity differences, and no taxa remained significant after false-discovery-rate correction. **Conclusions**: Crude beta-diversity differences between bovine milk consumers and plant-based beverage consumers were small and were not reproduced in propensity score-matched sensitivity analyses. These findings should be interpreted cautiously because of categorical exposure assessment, residual imbalance, limited PBBC sample size, dispersion differences in crude analyses, and potential unmeasured confounding.

## 1. Introduction

Diet is one of the major modifiable determinants of human health and exerts a substantial influence on metabolic, immune, and gastrointestinal homeostasis. Its biological effects depend not only on the nutritional composition of individual foods, but also on broader dietary patterns, lifestyle factors, environmental exposures, and host-related characteristics [1,2,3,4]. Within this complex framework, the gut microbiota has emerged as an important mediator of the relationship between diet and health. The intestinal microbial ecosystem contributes to nutrient metabolism, immune regulation, production of bioactive metabolites, and maintenance of intestinal barrier integrity [3,5,6]. Alterations in its composition and function have been associated with several chronic inflammatory and metabolic disorders, although the direction and clinical relevance of these associations often remain difficult to establish [3,5,6].

Dietary habits are among the main factors shaping gut microbiota composition and function. Both long-term dietary patterns and specific food components may influence microbial diversity, taxonomic structure, and metabolic activity [3,7]. However, the relationship between individual foods and the gut microbiota is complex because food choices are frequently associated with demographic, anthropometric, socioeconomic, and lifestyle characteristics that may independently affect microbial communities [1,2,3,8]. Consequently, associations identified in observational studies cannot always be interpreted as direct effects of a specific dietary exposure [1,2,8].

Milk and dairy products are widely consumed foods that provide high-quality proteins, lipids, vitamins, and minerals and contribute to the nutritional requirements of different population groups [9,10,11]. Their potential effects on the gut microbiota may be mediated by several components, including lactose, milk proteins, lipids, micronutrients, and bioactive peptides generated during digestion or processing [7,9,11]. Fermented dairy products may additionally influence the intestinal ecosystem by providing live microorganisms and fermentation-derived metabolites [12,13]. Available evidence suggests that dairy consumption may be associated with differences in gut microbial composition and cardiometabolic health; however, findings vary according to the type of dairy product, population characteristics, habitual diet, and analytical approach [7,12,13].

In parallel with conventional dairy consumption, plant-based beverages have become increasingly common as alternatives to bovine milk. These products are consumed for a variety of reasons, including dietary preferences, perceived health benefits, lactose intolerance, allergy-related concerns, and ethical or environmental considerations [9,10]. Nevertheless, plant-based beverages differ substantially from bovine milk in terms of protein quality, lipid profile, carbohydrate composition, fiber content, micronutrient fortification, and degree of processing [9,10]. Their consumers may also represent a distinct population subgroup characterized by different dietary habits, age distribution, body composition, and lifestyle patterns. Therefore, comparisons between bovine milk consumers, plant-based beverage consumers, and individuals who do not consume milk may reflect not only differences in the consumed products but also broader differences in the characteristics of the individuals selecting them [1,2,8].

In nutritional microbiome research, age, sex, adiposity, body composition, medication use, and overall dietary habits can influence gut microbiota composition and potentially confound associations attributed to single foods [1,2,3]. Therefore, analytical approaches that account for these factors are important when evaluating dietary–microbiota relationships [1,2,3,8].

The present analysis was conducted within a broader research project investigating commercial milk matrices and their chemical characteristics [14,15,16,17]. However, the current manuscript focuses exclusively on the cross-sectional association between questionnaire-derived milk consumption patterns and gut microbiota composition.

Previous studies have investigated the relationship between dairy intake and gut microbiota using both interventional and observational designs [17,18,19,20,21,22]. Although these studies support the biological plausibility of an association between dairy consumption and gut microbiota composition, findings remain heterogeneous because of differences in populations, exposure definitions, and analytical approaches. Importantly, most studies have evaluated dairy products as a broad exposure category rather than specifically comparing habitual bovine milk consumers, plant-based beverage consumers, and non-consumers. Consequently, direct evidence addressing these distinct consumption patterns remains limited, particularly because consumers of plant-based beverages may differ substantially from bovine milk consumers in demographic, anthropometric, and lifestyle characteristics that can independently influence gut microbiota composition [23].

To address this gap, the present study compared non-milk consumers, bovine milk consumers, and plant-based beverage consumers in a real-world cohort and evaluated whether observed microbiota differences persisted after accounting for major demographic and body composition imbalance through propensity score-matched sensitivity analyses. Although the cross-sectional design does not allow causal inference, it provides a useful framework to evaluate whether crude microbiota differences are robust to major measured sources of confounding.

## 2. Materials and Methods

### 2.1. Study Population

This observational cross-sectional study was conducted at the Section of Food Science, Clinical Nutrition and Pharmaceutical Science, Department of Biomedicine and Prevention, University of Rome Tor Vergata. The study protocol was approved by the Ethics Committee of the Calabria Region Center Area Section (Register Protocol No. 97, 20 April 2023).

All participants recruited into the study read and signed a written informed consent form in accordance with the 1975 Declaration of Helsinki, as revised in 2013. The study recruited a total of 265 participants with different environmental backgrounds, eating habits, and lifestyles. All enrolled subjects underwent a comprehensive assessment, which included the evaluation of their medical history, lifestyle habits, dietary patterns, nutritional status, and gut microbiota composition. No formal a priori sample size calculation was performed as this study represents an exploratory cross-sectional analysis of participants recruited within a broader research project. The final sample size was therefore determined by the number of eligible individuals available during the recruitment period.

### 2.2. Baseline Exposure Classification and Analytical Framework

Milk consumption habits were assessed at baseline, with the assistance of a trained operator, using a structured, self-reported questionnaire that was specifically developed to classify participants according to their usual milk consumption behavior. The questionnaire was completed during the clinical visit as part of a broader assessment of dietary habits. A trained operator was present to ensure that the questions were correctly understood and that responses were recorded consistently. Participants were asked to report on their long-standing consumption habits, referring to their usual behavior over the preceding years rather than occasional or recent consumption. The complete questionnaire and the criteria used to derive the analytical exposure groups are provided in Supplementary File S1.

Questionnaire responses were translated into two database fields: ‘milk consumption status’ and ‘vegetable milk’. These were used to derive the analytical exposure groups. Participants who reported no habitual milk consumption were classified as non-milk consumers (NMC; database label: NO_MILK). Among participants reporting habitual milk consumption, those with ‘vegetable milk’ equal to ‘NO’ were classified as bovine milk consumers (BMC; database label: MILK), whereas those with ‘vegetable milk’ equal to ‘YES’ were classified as plant-based beverage consumers (PBBC; database label: VEG_MILK). This classification generated three mutually exclusive analytical groups: NMC, BMC, and PBBC.

These questionnaire-derived exposure groups should be interpreted as coarse categorical proxies of long-standing habitual milk consumption patterns rather than as quantitative dietary exposure measures. Although categorical information on consumption frequency and usual portion size was collected for participants reporting bovine milk consumption, equivalent quantitative information was not available for plant-based beverage consumers or non-milk consumers. Therefore, intake amount, dose–response relationships, duration of exposure, plant-based beverage formulation, fortification, added sugar content, fermentation status, nutrient composition, and concurrent consumption of other dairy products could not be systematically evaluated. Accordingly, exposure misclassification and within-group heterogeneity cannot be excluded.

Unanswered frequency or portion-size items were treated as missing and were not imputed. The analytical framework was organized into five modules. The primary analysis compared NMC, BMC, and PBBC. The main secondary analysis involved a direct comparison of BMC and PBBC. Propensity score-matched analyses were performed as sensitivity analyses to assess the influence of demographic, anthropometric, and body composition imbalance. Brand-level and milk-only product-characteristic analyses were considered exploratory and reported as supplementary analyses.

### 2.3. Gut Microbiota Profiling and Data Processing

Stool samples were collected from all participants to profile their gut microbiota. Each participant received a standardized collection kit, along with written and oral instructions on how to collect, store, and transport the samples. Participants self-collected their samples at home, taking care to avoid contact with urine, toilet water, or other potential contaminants. The samples were then transported under controlled conditions to Wellmicro^®^ (Via Antonio Canova 30, 40138 Bologna, Italy), where DNA extraction, sequencing, and primary bioinformatic processing were performed.

Fecal DNA was extracted and analyzed using high-throughput sequencing of the bacterial 16S rRNA gene. Next-generation sequencing was performed using the Ion S5 platform (Thermo Fisher Scientific, Waltham, MA, USA). The resulting sequences were compared with curated bacterial reference databases, including Curated Greengenes v13.5 and the Curated MicroSEQ^®^ 16S Reference Library (Thermo Fisher Scientific, 2013) [24]. Sequence processing and taxonomic classification were conducted using the QIIME suite [25] and Ion Reporter software 5.20 (Thermo Fisher Scientific), according to the workflow adopted by the external laboratory.

Microbial community profiles were generated by taxonomic annotation of sequencing-derived microbial features. Taxonomic labels were assigned by the external laboratory on the basis of sequence similarity to the reference databases. Sequences showing ≥99% similarity were reported by the laboratory with species-level annotations when available, whereas sequences with 97–99% similarity were generally assigned at the genus level and those with lower similarity at higher taxonomic levels. However, because 16S rRNA sequencing does not consistently provide sufficient resolution for definitive species-level identification, species-level annotations were considered putative and interpreted cautiously. The composition of the gut microbiota was expressed as the relative abundance of each bacterial group, calculated as a percentage of the total microbial community identified in each sample.

Following primary processing by the external laboratory, the authors received a participant-by-taxon relative abundance matrix for downstream statistical and bioinformatic analyses. Each row corresponded to one participant and each column corresponded to an annotated microbial taxon or feature provided by the external laboratory. The matrix used for statistical analysis was identified in the cleaned dataset starting from the first microbiota abundance column and included all subsequent taxon-abundance variables. The authors did not perform de novo sequencing, OTU/ASV generation, or primary taxonomic assignment, but performed downstream statistical analyses on the abundance profiles supplied by the external laboratory.

Before statistical analysis, taxon-abundance variables were converted to numeric format. Blank or missing abundance cells in the supplied abundance matrix were audited before analysis and were represented as zero only when required for diversity and distance-based calculations as a computational representation of non-detected or blank abundance values in the provided table. Samples with unavailable microbiota profiles were not imputed. Alpha diversity was calculated on the taxon abundance matrix using richness, Shannon diversity, Simpson diversity, and Pielou evenness indices. Beta diversity was assessed using Bray–Curtis dissimilarity calculated on log1p-transformed abundance values and visualized through principal coordinate analysis. Taxa-level comparisons were also performed on log1p-transformed abundance values.

### 2.4. Statistical Analysis

All statistical analyses were performed using a modular R Markdown pipeline in R version 4.5.1 (13 July 2025). Data import, cleaning, and manipulation were performed using the readxl, janitor, dplyr, tidyr, stringr, purrr, forcats, and tibble packages. Statistical modeling and result extraction were supported by the stats, broom, and rstatix packages. Microbiota alpha- and beta-diversity analyses, including Bray–Curtis distance calculation and PERMANOVA, were performed using the vegan package. Propensity score matching and covariate balance diagnostics were performed using the MatchIt and cobalt packages. Figures were generated using ggplot2, while tables and Excel outputs were produced using knitr, kableExtra, and openxlsx. The analytical groups defined in Section 2.2 were used throughout all analyses: non-milk consumers (NMC), bovine milk consumers (BMC), and plant-based beverage consumers (PBBC). Continuous variables were summarized as mean, standard deviation, median, and interquartile range (IQR), while categorical variables were summarized as absolute and relative frequencies. Normality was assessed within each analytical group using the Shapiro–Wilk test. For two-group comparisons, normally distributed continuous variables were compared using Student t-tests, whereas non-normally distributed variables were compared using Wilcoxon rank-sum tests. For comparisons involving three or more groups, ANOVA or Kruskal–Wallis tests were used according to distributional assumptions. Categorical variables were compared using chi-square tests or Fisher exact tests when expected cell counts were small.

Alpha-diversity metrics were compared using Wilcoxon tests for binary contrasts and Kruskal–Wallis tests for multi-group comparisons. Beta diversity was assessed primarily using Bray–Curtis dissimilarity calculated on log1p-transformed abundance values. Differences in overall microbiota composition were tested by permutational multivariate analysis of variance (PERMANOVA) with 999 permutations. Homogeneity of multivariate dispersion was assessed using PERMDISP on the same distance matrices used for PERMANOVA. Sensitivity analyses were conducted using raw Bray–Curtis, binary Jaccard, and Aitchison/CLR-based distances to evaluate the robustness of beta-diversity results to alternative distance definitions and to the compositional nature of relative abundance data. Taxon-level analyses were performed on log1p-transformed abundance values using Wilcoxon tests for two-group comparisons and Kruskal–Wallis tests for multi-group comparisons.

The statistical framework was structured into five modules, as summarized in Table 1. The primary analysis compared non-milk consumers (NMC), bovine milk consumers (BMC), and plant-based beverage consumers (PBBC). The main secondary analysis directly compared BMC and PBBC. Pairwise propensity score-matched analyses were performed as sensitivity analyses to evaluate the influence of demographic, anthropometric, and body composition imbalance. Brand-level analyses among bovine milk consumers and milk-only product-characteristic models were considered exploratory and reported as supplementary analyses. In the multivariable exploration module, marginal PERMANOVA was used for beta diversity, and a multivariable linear model was fitted for Shannon diversity.

Pairwise propensity score matching was used as an exploratory sensitivity analysis. Propensity scores were estimated using logistic regression, including age, birth sex, BMI, WHR, and one selected non-collinear body composition parameter, chosen according to biological relevance, completeness, and collinearity diagnostics. Nearest neighbor 1:1 matching was performed without replacement using a logit propensity score distance and a caliper of 0.2 standard deviations of the logit of the propensity score. Participants with missing values in the exposure variable or in any covariate included in a given model were excluded from that matched comparison using a complete-case approach. Balance was evaluated using standardized mean differences, with |SMD| < 0.10 considered optimal. For matched beta-diversity analyses, PERMANOVA was stratified by matched pair/subclass.

Multiple testing was controlled using false discovery rate (FDR) correction for metadata and taxa-level analyses. For taxa-level analyses, FDR correction was applied within each predefined analytical module; taxa-level findings were therefore interpreted as exploratory unless they were consistent across sensitivity analyses.

## 3. Results

The final analytical cohort included 265 participants with available baseline information on milk consumption status and gut microbiota composition. Participants were classified into three questionnaire-derived milk consumption groups: non-milk consumers, bovine milk consumers, and plant-based beverage consumers. Bovine milk consumers represented the largest group, whereas plant-based beverage consumers accounted for approximately 10% of the cohort. The operational definition and sample size of each exposure group are reported in Table 2.

### 3.1. Global Comparison Among Non-Milk Consumers, Bovine Milk Consumers, and Plant-Based Beverage Consumers

**Figure 1:**
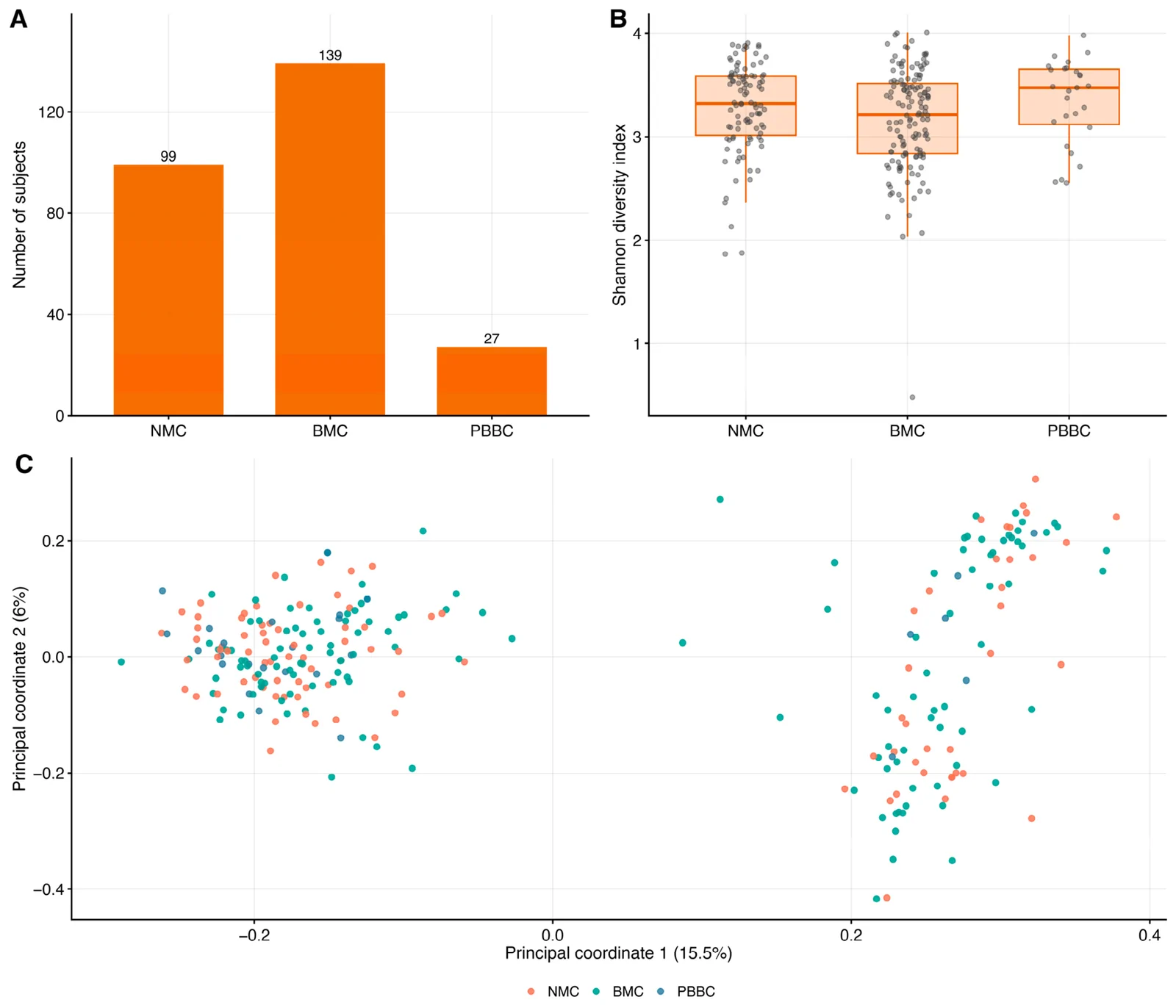
Global microbiota analysis according to milk consumption pattern. Participants were classified as non-milk consumers (NMC, n = 99), bovine milk consumers (BMC, n = 139), or plant-based beverage consumers (PBBC, n = 27). (**A**) Number of subjects in each exposure group. (**B**) Shannon alpha-diversity index across groups; overall Kruskal–Wallis test: *p* = 0.058, Benjamini–Hochberg-adjusted *q* = 0.116. (**C**) Bray–Curtis principal coordinate analysis based on log1p-transformed taxa abundances. PERMANOVA showed a small group-related component of variation (R^2^ = 0.0107, *p* = 0.050), while PERMDISP was significant (*p* = 0.006).

The primary analysis compared non-milk consumers (NMC), bovine milk consumers (BMC), and plant-based beverage consumers (PBBC). The analytical cohort included 265 participants classified into these three exposure groups: NMC (n = 99), BMC (n = 139), and PBBC (n = 27) (Table 3 and Figure 1A). The PBBC group represented approximately 10% of the cohort and was therefore substantially smaller than the other two groups, a factor that should be considered when interpreting multi-group comparisons.

In the primary analysis, Shannon alpha diversity did not differ significantly among the NMC, BMC, and PBBC groups (Kruskal–Wallis *p* = 0.058; Benjamini–Hochberg-adjusted *q* = 0.116; Figure 1B). Pairwise comparisons were also non-significant after Benjamini–Hochberg adjustment. Bray–Curtis PERMANOVA showed only a small group-related component of variation (R^2^ = 0.0107, *p* = 0.050; Figure 1C). However, PERMDISP was significant (*p* = 0.006), indicating that the observed beta-diversity pattern may be influenced by differences in multivariate dispersion rather than reflecting only a shift in group centroids.

**Figure 2:**
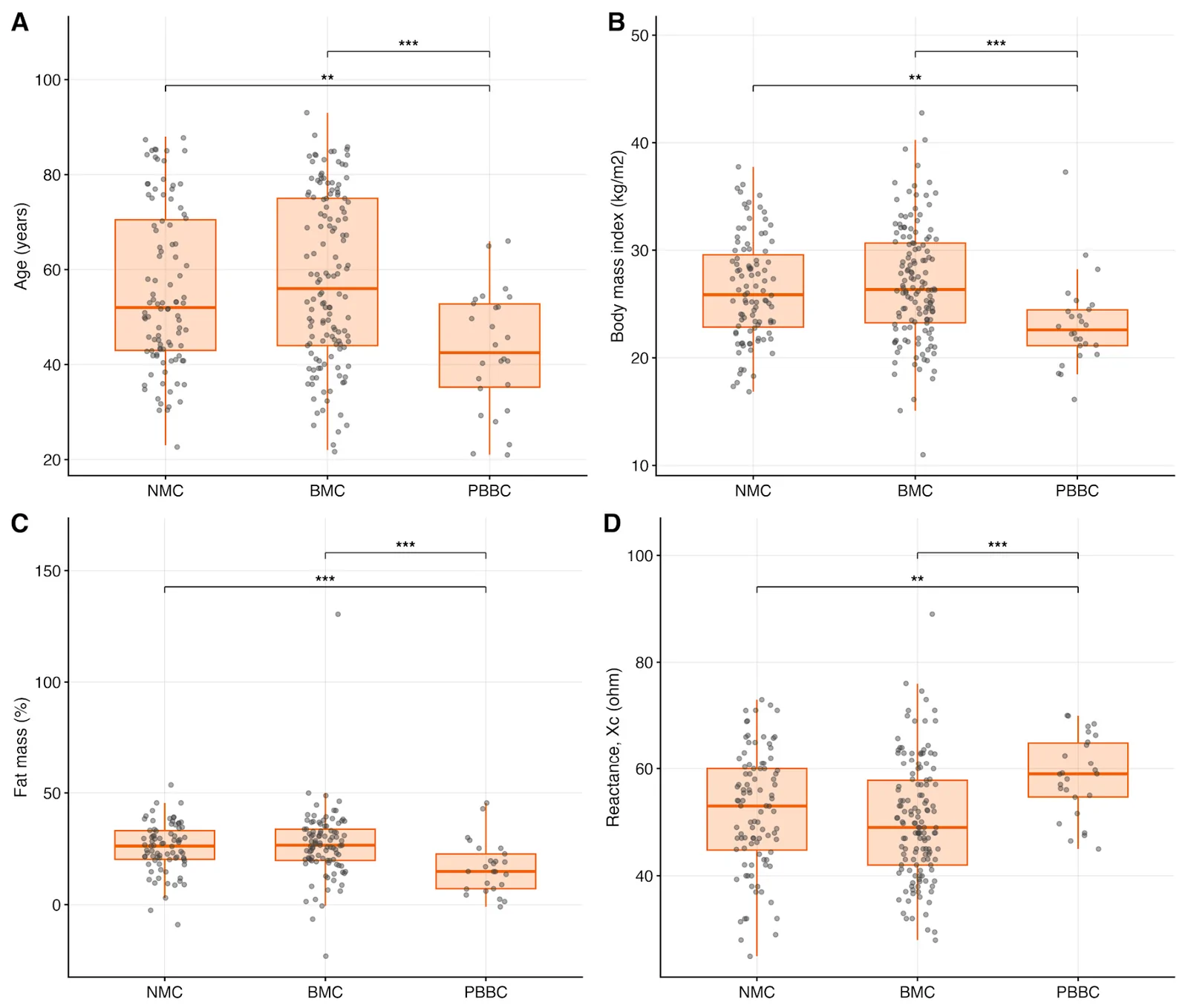
Baseline clinical and body composition differences across milk consumption groups. Participants were classified as non-milk consumers (NMC, n = 99), bovine milk consumers (BMC, n = 139), or plant-based beverage consumers (PBBC, n = 27). (**A**) Age, (**B**) body mass index, (**C**) fat mass percentage, and (**D**) reactance (Xc, ohm) differed significantly across groups by overall group-comparison tests, selected according to distributional assumptions. Asterisks indicate significant pairwise Wilcoxon comparisons after Benjamini–Hochberg adjustment: ** *p* < 0.01; *** *p* < 0.001.

In contrast to the microbiota diversity metrics, several baseline clinical and body composition variables differed significantly across exposure groups after FDR correction. Age, BMI, fat mass percentage according to De Lorenzo, and reactance (Xc, ohm) were significantly different across groups (Figure 2). In particular, PBBC participants appeared younger and showed lower BMIs and fat mass percentages, whereas Xc was higher compared with the other groups. These differences indicate relevant clinical and body composition imbalances across milk consumption categories.

### 3.2. Direct Comparison Between Bovine Milk and Plant-Based Beverage Consumers

**Figure 3:**
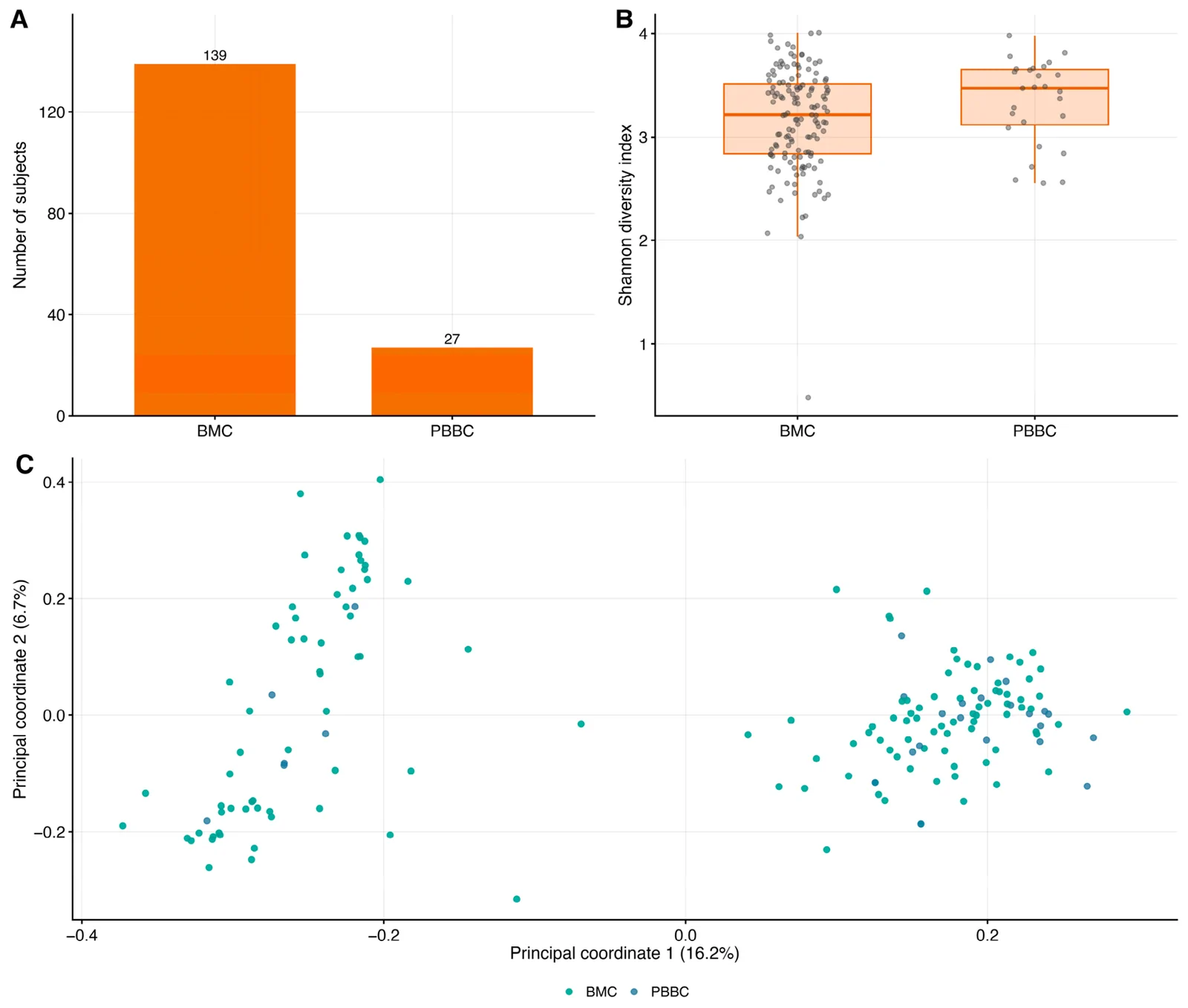
Microbiota comparison between bovine milk consumers and plant-based beverage consumers. Participants were classified as bovine milk consumers (BMC, n = 139) or plant-based beverage consumers (PBBC, n = 27). (**A**) Number of subjects in each group. (**B**) Shannon alpha diversity index. No statistically significant difference was observed between groups by Wilcoxon rank-sum test (*p* = 0.082; Benjamini–Hochberg-adjusted q = 0.142). (**C**) Bray–Curtis principal coordinate analysis based on log1p-transformed taxa abundances. PERMANOVA showed a small but statistically significant group-related component of variation (R^2^ = 0.0119, *p* = 0.014), while PERMDISP was also significant (*p* = 0.005), indicating that dispersion differences may contribute to the observed beta-diversity pattern.

The direct crude comparison between bovine milk consumers and plant-based beverage consumers included 166 participants: 139 BMC and 27 PBBC (Figure 3A). Shannon alpha diversity did not differ significantly between groups (Wilcoxon *p* = 0.082; Benjamini–Hochberg-adjusted *q* = 0.142; Figure 3B), although median values were numerically higher in PBBC. Bray–Curtis PERMANOVA was significant but explained only a small proportion of the variation (R^2^ = 0.0119, *p* = 0.014; Figure 3C). PERMDISP was also significant (*p* = 0.005), suggesting that differences in multivariate dispersion may contribute to the observed beta-diversity result. Therefore, the crude beta-diversity difference was interpreted cautiously and was further evaluated in matched sensitivity analyses.

**Figure 4:**
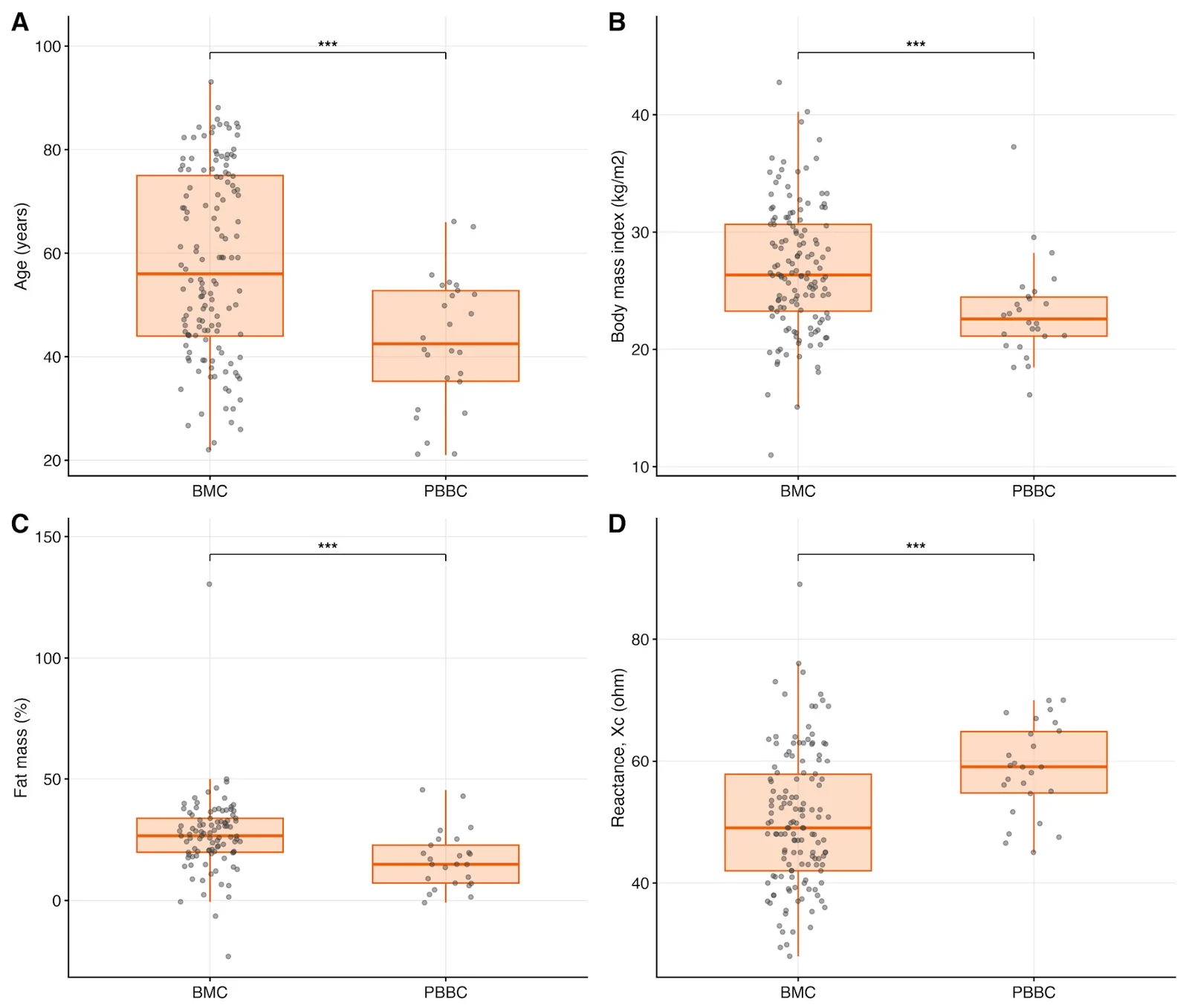
Baseline clinical and body composition differences between bovine milk consumers and plant-based beverage consumers. Participants were classified as bovine milk consumers (BMC, n = 139) or plant-based beverage consumers (PBBC, n = 27). (**A**) Age, (**B**) body mass index, (**C**) fat mass percentage, and (**D**) reactance (Xc, ohm) differed significantly between groups by two-group tests selected according to distributional assumptions, with *p* < 0.001 for all comparisons. Asterisks indicate statistical significance: *** *p* < 0.001.

At the taxon level, one feature remained significant after within-module FDR correction in the crude BMC versus PBBC comparison, with the top FDR-adjusted signal corresponding to *Tepidibaculum saccharolyticum*. However, no taxa/features remained significant after FDR correction in the matched analyses. Therefore, taxa-level findings were considered exploratory and were not interpreted as robust product-specific microbiota signatures. The number of tested features, q-values, prevalence estimates, median abundances, and effect-size estimates are reported in the Supplementary Materials. The same crude comparison also revealed marked differences in age, BMI, fat mass percentage, and Xc (Figure 4). These variables are biologically plausible confounders of microbiota composition and limit the interpretation of the crude beta-diversity signal as a direct milk consumption association. This imbalance motivated the subsequent propensity score-matched sensitivity analyses.

### 3.3. Propensity Score-Matched Sensitivity Analyses

To address the clinical and body composition imbalance observed in the crude comparisons, pairwise propensity score matching was performed as an exploratory sensitivity analysis using age, birth sex, BMI, WHR, and one selected non-collinear body composition parameter. Three matched comparisons were evaluated: non-milk consumers versus bovine milk consumers (NMC vs. BMC), non-milk consumers versus plant-based beverage consumers (NMC vs. PBBC), and bovine milk consumers versus plant-based beverage consumers (BMC vs. PBBC) (Table 4).

**Figure 5:**
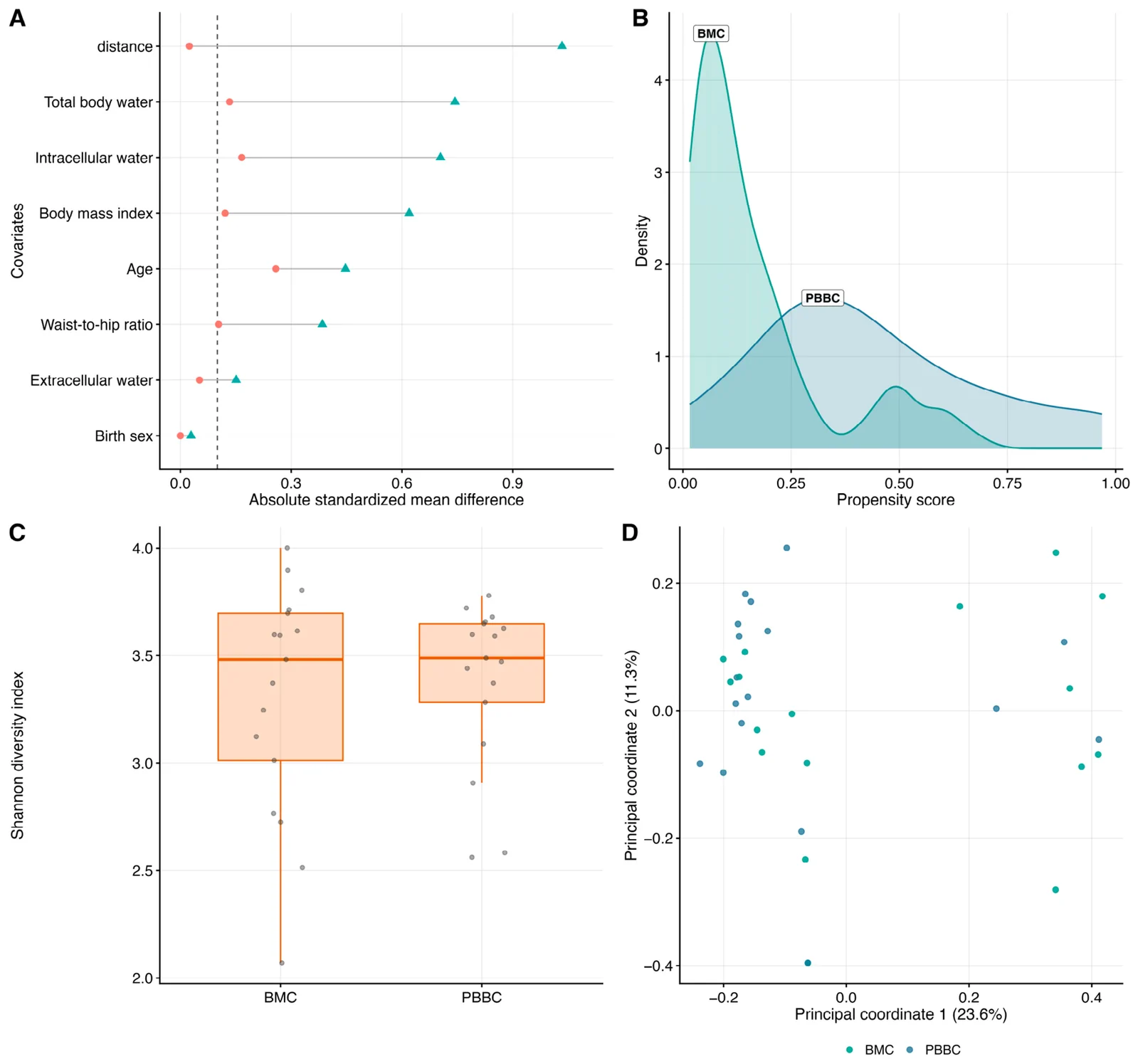
Propensity score-matched sensitivity analysis comparing bovine milk consumers and plant-based beverage consumers. Matched groups included bovine milk consumers (BMC, n = 23) and plant-based beverage consumers (PBBC, n = 23). (**A**) Absolute standardized mean differences before and after matching; the dashed line indicates the 0.10 balance threshold. (**B**) Pre-matching propensity score distribution. (**C**) Shannon alpha diversity after matching using a paired Wilcoxon signed-rank test (*p* = 0.475). (**D**) Bray–Curtis principal coordinate analysis based on log1p-transformed taxa abundances after matching; PERMANOVA was stratified by matched pair/subclass (R^2^ = 0.0214, *p* = 0.506), and PERMDISP was not significant (*p* = 0.582).

In matched sensitivity analyses, no pairwise comparison retained significant alpha-diversity, beta-diversity, or taxa-level differences after FDR correction. For the BMC versus PBBC matched subset, 23 matched pairs were retained. The paired Shannon comparison was not significant (*p* = 0.475), and pair-stratified PERMANOVA did not indicate a significant beta-diversity difference (R^2^ = 0.0214, *p* = 0.506; Figure 5C,D). PERMDISP was not significant in this matched subset (*p* = 0.582). No taxa remained significant after FDR correction in any matched comparison.

Matching diagnostics indicated that covariate balance improved after matching but was not optimal in all comparisons. The NMC vs. BMC comparison achieved the best post-matching balance among the three pairwise analyses, with a maximum absolute standardized mean difference after matching of 0.128. The NMC vs. PBBC comparison also showed improved balance, with a maximum post-matching |SMD| of 0.141. By contrast, residual imbalance remained more evident for the BMC vs. PBBC comparison, with a maximum post-matching |SMD| of 0.262. Propensity score density plots also showed incomplete overlap, particularly for comparisons involving PBBC. Therefore, matched analyses should be interpreted as exploratory sensitivity analyses rather than definitive causal estimates.

As an exploratory supplementary analysis, we investigated the visible clustering observed in the PCoA plots, which was not fully aligned with exposure groups. Associations between ordination coordinates and available metadata were explored. PC1 and PC2 were associated with several body composition variables and dominant microbial features, suggesting that the main ordination structure was not exclusively driven by milk consumption groups. Dedicated sequencing batch variables, such as sequencing run, extraction batch, read depth, or sample processing date, were not available in the dataset and therefore could not be formally tested.

Finally, exploratory analyses restricted to bovine milk consumers did not provide evidence of robust global microbiota differences related to product-level characteristics, including milk brand, milk category, processing type, or specific milk type. These analyses were therefore considered supplementary and are not discussed as primary findings.

## 4. Discussion

This study provides an integrative and methodologically cautious assessment of the relationship between milk consumption patterns and gut microbiota composition in a cohort of healthy adults. The principal finding was that crude microbiota differences between bovine milk consumers and plant-based beverage consumers were small and were not reproduced in propensity score-matched sensitivity analyses. In the primary three-group analysis, Shannon alpha diversity did not differ significantly among non-milk consumers, bovine milk consumers, and plant-based beverage consumers. Bray–Curtis PERMANOVA showed only a small group-related component of variation, and the significant PERMDISP result indicated that differences in multivariate dispersion may have contributed to the observed beta-diversity pattern. Similarly, in the crude BMC versus PBBC comparison, Bray–Curtis PERMANOVA was statistically significant but explained a limited proportion of variation, while PERMDISP was also significant. Therefore, the crude beta-diversity findings should be interpreted cautiously and should not be considered definitive evidence of a direct product-related difference in gut microbiota composition.

Previous studies have reported associations between dairy intake and gut microbiota composition, often considering dairy products as a broad exposure category or focusing on specific populations and fermented products. The present study differs by directly comparing non-milk consumers, bovine milk consumers, and plant-based beverage consumers within the same cohort and by explicitly evaluating whether crude microbiota differences persisted after propensity score-matched sensitivity analyses. In this context, the crude BMC versus PBBC beta-diversity result was not reproduced in the matched subset. This observation suggests that participant-level demographic, anthropometric, and body composition differences may contribute to unadjusted microbiota patterns. However, because of the cross-sectional design, categorical exposure assessment, residual imbalance, and unmeasured confounding, this finding should be interpreted as hypothesis-generating rather than as evidence against a biological relationship between milk-related exposures and the gut microbiota.

These results highlight a central methodological challenge in nutritional microbiome research. Food choices rarely occur in isolation and are commonly embedded within broader behavioral, demographic, and metabolic profiles. Individuals selecting plant-based beverages may differ from bovine milk consumers not only in the type of beverage consumed, but also in age, adiposity, dietary pattern, health awareness, ethical preferences, physical activity, and other lifestyle factors. Consequently, an association attributed to a single food or beverage may partly reflect the characteristics of the population choosing that product. In the present study, PBBC participants differed from BMC participants in several baseline characteristics, including age, BMI, fat mass percentage, and impedance-derived parameters. These differences are biologically relevant because age, adiposity, and body composition characteristics are known determinants of gut microbiota composition and may also be associated with broader dietary and lifestyle patterns.

The matched analyses were designed to explore whether the crude microbiota findings were robust to adjustment for measured demographic, anthropometric, and body composition imbalance. In the BMC versus PBBC matched subset, the paired Shannon comparison was not significant, pair-stratified PERMANOVA did not indicate a significant beta-diversity difference, and PERMDISP was not significant. However, these findings should not be interpreted as definitive evidence that no independent association exists. The matched analyses were limited by reduced sample size, incomplete propensity score overlap, and residual imbalance, particularly in comparisons involving PBBC. Therefore, the matched findings should be regarded as exploratory sensitivity analyses rather than causal estimates.

Taxa-level findings also require cautious interpretation. In the crude BMC versus PBBC comparison, one feature remained significant after within-module FDR correction. However, no taxa or features remained significant after FDR correction in the matched analyses. Therefore, taxa-level findings were considered exploratory and were not interpreted as robust product-specific microbiota signatures. The number of tested features, q-values, prevalence estimates, median abundances, and effect-size estimates are reported in the Supplementary Materials. This approach is particularly important because microbiota abundance data are high-dimensional and compositional, and individual taxon-level signals may be influenced by multiple testing, dispersion effects, residual confounding, or chance findings.

The present findings should also be considered in relation to previous dairy–microbiota studies. Several investigations have described associations between dairy intake and gut microbiota diversity, taxonomic structure, or cardiometabolic profiles. However, many previous studies evaluated dairy products as a broad category, often combining milk with yogurt, cheese, fermented milk, kefir, or other dairy derivatives. Fermented dairy products may influence the intestinal ecosystem through live microorganisms and fermentation-derived metabolites, whereas non-fermented bovine milk and plant-based beverages differ substantially in protein quality, carbohydrate composition, lipid profile, fiber content, micronutrient fortification, and processing. In addition, previous studies often focused on selected populations or specific dietary interventions, whereas the present analysis evaluated questionnaire-derived habitual consumption patterns in a real-world cross-sectional cohort. These differences in study design, exposure definition, and population characteristics may explain why the results are not directly comparable across studies. The absence of robust associations after matching should not be interpreted as evidence that milk consumption patterns are biologically irrelevant to the gut microbiota. Rather, any independent association may be modest, dependent on specific product characteristics, or detectable only when exposure is characterized more precisely. The current classification did not fully capture quantitative intake, portion size, duration of consumption, type of plant-based beverage, fortification, added sugars, fat content, fermentation status, or concurrent consumption of other dairy products. Consequently, the present study should be interpreted as an evaluation of broad habitual milk consumption patterns rather than a dose–response analysis or a product-specific nutritional investigation.

Exploratory analyses restricted to bovine milk consumers did not provide evidence of robust global microbiota differences related to product-level characteristics, including milk brand, milk category, processing type, or specific milk type. These analyses should therefore be considered hypothesis-generating and are more appropriately reported in the Supplementary Materials rather than emphasized as primary findings. The present analysis forms part of a broader multidisciplinary project investigating commercial milk matrices, including their chemical characterization and the potential biological relevance of food-associated contaminants. However, the current study exclusively evaluates baseline observational data and does not include individual contaminant exposure, chemical measurements of the products habitually consumed by each participant, or cellular outcomes. The findings therefore cannot be used to draw conclusions regarding the effects of contaminants or chemically characterized milk matrices. These components will require separate analyses specifically designed to link product-level chemical data with biological endpoints.

Several limitations should be considered when interpreting these findings. First, the cross-sectional design precludes causal inference and does not allow reverse causation to be excluded. Pre-existing gastrointestinal tolerance, lactose intolerance, or malabsorption may have influenced participants’ habitual choice to consume, avoid, or replace bovine milk, and gut microbiota composition may itself be related to such tolerance. Second, exposure assessment was categorical and self-reported; therefore, quantitative intake, portion size, duration of consumption, plant-based beverage formulation, fortification, added sugar, fermentation status, and concurrent consumption of other dairy products were not fully captured. Third, the PBBC subgroup was small, resulting in limited statistical power, reduced matched sample size, incomplete propensity score overlap, and residual covariate imbalance. Fourth, propensity score matching can account only for measured covariates. Residual confounding by overall diet quality, energy and fiber intake, fermented foods, probiotic use, medication and antibiotic exposure, smoking, alcohol intake, physical activity, socioeconomic status, geography, seasonality, and technical sequencing factors cannot be excluded. Fifth, the taxon-level analyses were exploratory and should be interpreted in light of the compositional nature of microbiota data and the large number of statistical comparisons. In particular, the single FDR-significant feature observed in the crude BMC versus PBBC comparison was not reproduced in matched analyses and should therefore be regarded as hypothesis-generating only. Finally, species-level taxonomic assignments derived from 16S rRNA sequencing should be interpreted cautiously because this approach may not provide sufficient resolution to reliably distinguish closely related bacterial species. Potential technical sources of variation, including sample processing and sequencing-related batch effects, should also be considered. Although exploratory PCoA-driver analyses suggested that the main ordination structure was associated with body composition variables and dominant microbial features, dedicated technical batch variables, including sequencing run, extraction batch, read depth, and sample processing date, were not available and therefore could not be formally tested. Despite these limitations, this study has several strengths. The overall cohort was relatively large for an exploratory observational microbiome analysis, and detailed anthropometric and body composition measurements were available. The analytical strategy explicitly compared crude and matched results rather than relying exclusively on unadjusted group comparisons. The use of false discovery rate correction, PERMDISP, propensity score diagnostics, pair-stratified matched analyses, and transparent reporting of residual imbalance reduced the risk of overinterpreting nominally significant findings. Importantly, this study illustrates how crude microbiota associations in nutritional observational studies may be sensitive to participant-level differences and to the analytical handling of dispersion, matching, and multiple testing.

Future studies should adopt prospective or interventional designs, recruit adequately sized and better-balanced exposure groups, and collect detailed quantitative information on both milk and overall dietary intake. Product-level characterization should distinguish bovine milk from fermented dairy products and should classify plant-based beverages according to their botanical source, formulation, fortification, sugar content, and processing. Integration of these data with microbial functional profiling, metabolomics, and chemical characterization of the consumed products would help clarify whether specific milk-related components are associated with reproducible microbiota responses.

## 5. Conclusions

In this cross-sectional cohort, crude beta-diversity differences between bovine milk consumers and plant-based beverage consumers were small and were not reproduced in propensity score-matched sensitivity analyses. However, categorical exposure assessment, residual imbalance, limited sample size in the PBBC group, significant dispersion differences in crude analyses, and potential unmeasured confounding preclude definitive conclusions regarding the absence of an independent biological association. Larger prospective and interventional studies integrating detailed dietary assessment with product-level nutritional and chemical characterization are needed to determine whether specific characteristics of bovine milk or plant-based beverages exert independent effects on the gut microbiota.

## Figures and Tables

**Table 1:** Analytical modules and statistical purpose.

Module	Population	Comparison	Main Purpose	Core Methods
M1	Overall cohort	NMC vs. BMC vs. PBBC	Primary global comparison	Metadata tests; alpha diversity; PERMANOVA; taxa-level FDR
M2	Drinkers only	BMC vs. PBBC	Animal vs. plant-based milk contrast	Two-group metadata tests; alpha diversity; PERMANOVA; taxa-level FDR
M3	Bovine milk consumers	Product brand groups	Brand-level heterogeneity	Brand comparisons; alpha diversity; PERMANOVA; taxa-level FDR
M4	Bovine milk consumers	Milk-related product characteristics	Independent product predictors	Marginal PERMANOVA; linear model on Shannon
M5	Matched subsets	Pairwise contrasts among analytical groups	Sensitivity analysis for confounding	Propensity score matching; balance diagnostics; alpha diversity; PERMANOVA; taxa-level FDR

**Table 2:** Participants were classified as non-milk consumers (NMC), bovine milk consumers (BMC), or plant-based beverage consumers (PBBC) according to their self-reported habitual milk consumption patterns. Percentages are relative to the total analytical cohort.

Exposure Group	Definition	*n* (%)
NMC	milk_consumption_status = NO	99 (37.4)
BMC	milk_consumption_status = YES and vegetable_milk = NO	139 (52.5)
PBBC	milk_consumption_status = YES and vegetable_milk = YES	27 (10.2)

**Table 3:** NMC, non-milk consumers; BMC, bovine milk consumers; PBBC, plant-based beverage consumers; FDR, false discovery rate; PERMANOVA, permutational multivariate analysis of variance.

Module	Comparison	Shannon p	PERMANOVA R^2^	PERMA- NOVA *p*	Taxa with FDR-Adjusted *p* < 0.05	Main Finding
M1	NMC vs. BMC vs. PBBC	0.058	0.0107	0.050	0	Shannon diversity did not differ significantly across groups. Bray–Curtis PERMANOVA showed only a small group-related component of variation; however, PERMDISP was significant, indicating that dispersion differences may contribute to the beta-diversity pattern.
M2	BMC vs. PBBC (crude comparison)	0.082	0.0119	0.014	1	A small crude beta-diversity difference was observed between BMC and PBBC. However, PERMDISP was significant, and groups differed clinically and anthropometrically; therefore, this result was interpreted cautiously and evaluated in matched sensitivity analyses.
M3	Bovine milk brand analysis	0.601	0.0393	0.275	9	No significant global brand-level beta-diversity separation was observed. Taxa-level findings were considered exploratory because brand groups were unbalanced and the global beta-diversity test was not significant.
M4	Milk-only multivariable model	—	—	0.047	—	One nominal exploratory beta-diversity association was observed in the milk-only model, but this analysis was considered supplementary and was not interpreted as a robust independent predictor analysis.
M5	Propensity score-matched comparisons	0.475–0.817	0.0080–0.0246	0.223–0.506	0	No matched comparison retained significant alpha-diversity, beta-diversity, or taxa-level differences after FDR correction. Matched analyses were interpreted as exploratory sensitivity analyses because of reduced sample size, incomplete overlap, and residual imbalance.

**Table 4:** Propensity score-matched sensitivity analyses. Pairwise propensity score matching was performed as an exploratory sensitivity analysis. PERMANOVA analyses were stratified by matched pair/subclass. No matched comparison retained significant alpha-diversity, beta-diversity, or taxa-level differences after FDR correction. Residual imbalance and incomplete propensity score overlap remained, particularly in comparisons involving PBBC.

Matched Comparison	Complete Cases Before Matching	Matched Sample	Max |SMD| Pre → Post	Shannon *p*	PERMANOVA R^2^; *p*	PERMDISP p	Taxa FDR < 0.05
NMC vs. BMC	79 vs. 96	73 vs. 73	0.370 → 0.128	0.817	0.0080; *p* = 0.223	0.551	0
NMC vs. PBBC	79 vs. 26	23 vs. 23	0.789 → 0.141	0.749	0.0246; *p* = 0.322	0.599	0
BMC vs. PBBC	96 vs. 26	23 vs. 23	1.033 → 0.262	0.475	0.0214; *p* = 0.506	0.582	0

## Data Availability

The data presented in this study are available on request from the corresponding author. The data are not publicly available due to privacy.

## Supplementary Materials

The following supporting information can be downloaded at: https://www.mdpi.com/article/10.3390/nu18193272/s1, File S1: Questionnaire used for the assessment and classification of habitual milk consumption behavior. This structured questionnaire was specifically developed for the project and was completed during the baseline clinical visit as an interviewer-assisted self-report instrument. Participants were instructed to answer with reference to their usual, long-standing consumption habits over the preceding years rather than occasional or recent consumption.
