# Supplementary material for: Milk Consumption Patterns and Gut Microbiota Composition in Healthy Adults: Insights from a Propensity Score-Matched Cross-Sectional Study
Source: Nutrients. 2026 Oct 5;18(19):3272. doi: 10.3390/nu18193272 (PMC13667258; doi:10.3390/nu18193272)
Supplement: Supplementary file 1 [file nutrients-18-03272-s001.zip › nutrients-4595478-File S1.pdf]

Item	Question	Response options
1	Do you habitually consume milk?	☐ Yes<br>☐ No—proceed to Questions 11 and 12
2	Which type of milk do you usually consume?	☐ Bovine milk<br>☐ High-digestibility bovine milk<br>☐ Lactose-free milk<br>☐ Fortified bovine milk (e.g., enriched with vitamin D or folic acid)<br>☐ Sheep milk<br>☐ Goat milk<br>☐ Donkey milk<br>☐ Buffalo milk<br>☐ Mixed milk
3	If you consume lactose-free milk, what is the main reason?	☐ I prefer its taste or sensory characteristics<br>☐ I digest it better<br>☐ I believe I am lactose intolerant<br>☐ I have a diagnosis of lactose intolerance or lactose malabsorption<br>☐ Other: ____________________
Instruction	The following questions refer specifically to bovine milk.	—
4	How frequently do you consume milk?	☐ Daily<br>☐ Weekly<br>☐ Occasionally<br>☐ Never
5	Which fat-content category do you consume most frequently?	☐ Whole milk<br>☐ Semi-skimmed milk<br>☐ Skimmed milk
6	Do you preferentially consume organic or conventionally produced milk?	☐ Organic<br>☐ Conventional
7	What is your usual milk portion size?	☐ One espresso cup (approximately 50 mL)<br>☐ One glass (approximately 125 mL)<br>☐ One cup (approximately 250 mL)<br>☐ Other, please specify: ____________________
8	Which type of milk processing or preservation do you usually choose?	☐ Fresh pasteurised milk<br>☐ Long-life milk<br>☐ UHT milk<br>☐ Microfiltered milk<br>☐ Other: ____________________<br>☐ I do not know
9	Which milk brand do you usually consume?	☐ Parmalat<br>☐ Granarolo<br>☐ Accadì<br>☐ Latte Sano<br>☐ Centrale del Latte<br>☐ Other, please specify: ____________________

10	At what temperature do you usually consume milk?	☐ Cold<br>☐ Warm (e.g., heated in a microwave oven)<br>☐ Very hot or nearly boiling (e.g., milk used for cappuccino)
11	If you do not consume milk, what is the main reason?	☐ I do not like it<br>☐ I have lactose intolerance or lactose malabsorption<br>☐ I am allergic to milk proteins
12	Do you consume plant-based beverages instead of, or as an alternative to, milk (e.g., oat, rice or coconut beverages)?	☐ Yes<br>☐ No
